# Citrate and low-dose heparin combined anticoagulation in pediatric continuous renal replacement therapy

**DOI:** 10.1038/s41598-024-64433-6

**Published:** 2024-06-12

**Authors:** Desheng Zhu, Jie He, Zhenghui Xiao, Xiong Zhou, Xinping Zhang

**Affiliations:** Pediatric Intensive Care Unit, The Affiliated Children’s Hospital of Xiangya School of Medicine, Central South University (Hunan Children’s Hospital), No. 86 Ziyuan Rd, Yuhua District, Changsha, 410007 Hunan China

**Keywords:** Continuous renal replacement therapy, Children, Regional citrate anticoagulation, Heparin, D-dimer, Continuous renal replacement therapy, Paediatric research

## Abstract

There remains no optimal anticoagulation protocol for continuous renal replacement therapy (CRRT) with regional citrate anticoagulation (RCA) in pediatric patients with elevated D-dimer levels. We aimed to assess the effects of different anticoagulation strategies on the risk of CRRT filter clotting in these patients. Pediatric patients undergoing CRRT were retrospectively grouped based on pre-CRRT D-dimer levels and anticoagulant: D-RCA group (normal D-dimer, RCA only, n = 22), D+ RCA group (elevated D-dimer, RCA only, n = 50), and D+ RCA+ systemic heparin anticoagulation (SHA) group (elevated D-dimer, RCA combined with SHA, n = 55). The risk of filter clotting and incidence of bleeding were compared among the groups. Among the groups, the D+ RCA+ SHA group had the longest filter lifespan; further, the incidence of bleeding was not increased by concurrent use of low-dose heparin for anticoagulation. Moreover, concurrent heparin anticoagulation was associated with a decreased risk of filter clotting. Contrastingly, high pre-CRRT hemoglobin and D-dimer levels and post-filter ionized calcium level > 0.4 mmol/L were associated with an increased risk of filter clotting. RCA combined with low-dose heparin anticoagulation could reduce the risk of filter clotting and prolong filter lifespan without increasing the risk of bleeding in patients with elevated D-dimer levels undergoing CRRT.

## Introduction

Effective anticoagulation is an essential requirement for ensuring smooth operation of continuous renal replacement therapy (CRRT). The overall goal of anticoagulation in CRRT is to maximize filter performance while reducing the complications associated with anticoagulation. Prolonging filter lifespan can enhance clearance efficiency, reduce blood loss in pediatric patients, lower healthcare costs, and alleviate medical staff workload^1–4^. Currently, systemic heparin anticoagulation (SHA) and regional citrate anticoagulation (RCA) are the two most widely used anticoagulation methods. Compared with SHA, RCA causes fewer bleeding complications and significantly prolongs the filter lifespan^5^. Therefore, RCA is the first-choice anticoagulation method in CRRT^6–9^. In clinical practice, poor anticoagulation effects have been observed following the adoption of the RCA regimen in pediatric patients with elevated D-dimer levels, leading to premature filter clotting despite achieving the target post-filter ionized calcium level and ultimately resulting in unplanned CRRT interruptions. Elevated D-dimer levels are negatively correlated with the CRRT filter lifespan^10^. In patients with elevated D-dimer levels, attempts to improve anticoagulation by reducing the blood flow rate and/or increasing citrate flow rate may not achieve the required effect. Instead, they may even increase the risk of complications. Therefore, there is an urgent need to determine suitable anticoagulation protocols for these patients. In theory, citrate and heparin anticoagulation have different targets of action. In patients at a high risk of hypercoagulability due to elevated D-dimer levels, a combination of citrate anticoagulation and low-dose heparin anticoagulation can achieve effective anticoagulation during CRRT without increasing the risk of bleeding and other complications, which helps prevent adverse reactions caused by excessive doses of a single anticoagulant. Currently, research on the combination of citrate and heparin anticoagulation during CRRT has mainly been limited to patients with coronavirus disease 2019 (COVID-19)^10,11^. Therefore, this study aimed to assess the effects of different anticoagulation strategies on the risk of CRRT filter clotting in patients with elevated D-dimer levels.

## Methods

### Participants

This retrospective single-center observational study included pediatric patients who underwent CRRT during hospitalization at the pediatric intensive care unit (PICU) of Hunan Children’s Hospital between March 2019 and February 2023. RCA is the first-choice anticoagulation regimen for CRRT at our unit, with the continuous veno-venous hemodiafiltration (CVVHDF) mode being mainly used during RCA. From March 2021 onwards, we attempted administering low-dose heparin anticoagulation in addition to RCA in pediatric patients with D-dimer levels > 0.5 μg/mL without contraindications to heparin.

The inclusion criteria were as follows: (1) anticoagulation was performed by RCA only or RCA combined with SHA and (2) CRRT was administered using the CVVHDF mode. The exclusion criteria were as follows: (1) death within 48 h after commencing CRRT; (2) cessation of CRRT due to non-catheter-related reasons (e.g., discharge against medical advice, death); (3) self-circulation during CRRT due to non-catheter-related reasons (e.g., out-of-hospital examinations); and (4) possibility of citrate metabolism disorder due to liver failure or refractory shock. Based on the pre-CRRT D-dimer levels and anticoagulation method, the patients were divided into the following three groups: those with normal D-dimer levels who underwent RCA only (D- RCA only group), those with elevated D-dimer levels who underwent RCA only (D+ RCA only group), and those with elevated D-dimer levels who underwent RCA combined with SHA (D+ RCA+ SHA group). In each patient, only the first CRRT session was analyzed, regardless of the number of times that the patient underwent CRRT during hospitalization in the PICU. Given that it was a retrospective observational study, the research content did not involve patient privacy or commercial interests, the requirement for informed consent was waived by the Ethics Committee of Hunan Children’s Hospital (approval no.: HCHLL-2023-192). This study followed the Strengthening the Reporting of Observational Studies in Epidemiology (STROBE) reporting guideline for cohort studies.

### CRRT anticoagulation strategies

A 4% anticoagulant citrate solution (Chengdu Qingshan Likang Pharmaceutical Co., Ltd., Sichuan, China) was used as the anticoagulant in all included patients. The initial blood pump flow rate was 3 mL/kg/min, with the initial citrate flow rate (mL/h) being 1.5× blood pump flow rate (mL/min). The target intracorporeal and post-filter calcium levels were 1–1.2 and 0.2–0.4 mmol/L, respectively. From March 2021 onwards, attempts were made to reduce the risk of clotting in the extracorporeal circuit during CRRT in patients with a high clotting risk due to elevated D-dimer levels. After excluding patients with contraindications to heparin and those receiving systemic anticoagulants (including heparin and low-dose heparin) due to other reasons, continuous intravenous infusion of 5 U/kg/h heparin with RCA was administered in patients with D-dimer levels > 0.5 μg/mL. The heparin dose was lowered if the activated partial thromboplastin time (aPTT) exceeded twice the normal aPTT values or the activated clotting time exceeded 180 s. Moreover, heparin was discontinued if the patient experienced a clinical bleeding episode during combination anticoagulation therapy.

### CRRT prescription

Indications for CRRT commencement were determined at the discretion of the attending physician at the PICU. CRRT was performed using the Prismaflex device (Gambro, Lund, Sweden), the multiFiltrate multifunctional blood purification system (Fresenius Medical Care, Bad Homburg, Germany), and the corresponding catheter and filter sets of the CRRT equipment. Vascular access was gained by placing a single dual-lumen catheter in the right internal jugular or femoral veins under ultrasound guidance. The initial dialysate and replacement fluid flow rates during CVVHDF were set at approximately 35 mL/kg/h, adopted post-dilution during the RCA-CVVHDF. Calcium-containing, bicarbonate-free hemofiltration and replacement fluid (4 L/bag; Chengdu Qingshan Likang Pharmaceutical Co., Ltd., Sichuan, China) was used as the replacement fluid. Bicarbonate supplementation was not performed during CRRT initiation. Buffer bases were generated after sodium citrate metabolism and adjusted based on the acid–base status of the patient. Calcium supplementation was performed based on intracorporeal ionized calcium; blood calcium levels were monitored during CRRT. Blood gas analysis, including measurement of ionized calcium and post-filter ionized calcium levels, was conducted once every 4–6 h. Routine blood tests, liver and kidney function tests, blood electrolyte level measurement, and comprehensive coagulation profiling were performed once every 6–8 h. Treatment parameters during CRRT were adjusted by titration based on the patient’s condition and monitored indicators.

### Data collection

Demographic characteristics, clinical data, CRRT parameters, and patient outcomes were recorded. Filter lifespan and the incidence of bleeding episodes during CRRT were set as the primary and secondary outcomes, respectively. A bleeding episode was defined as any clinically visible bleeding or a > 20 g/L decrease in hemoglobin levels during CRRT.

### Statistical methods

All statistical analyses were performed using SPSS 25.0 (IBM Corp., Armonk, NY). Normally distributed continuous variables are expressed as mean ± standard deviation (x ± s) and were compared using analysis of variance. Categorical data are expressed as counts (percentages) and were compared using the Chi-squared test. Non-normally distributed continuous data are expressed as median (quartiles) [*M (P25, P75)*] and were compared using the Kruskal–Wallis *H* test. Filter lifespan was analyzed using Kaplan–Meier estimates. Factors influencing filter clotting were analyzed using the Cox proportional hazards model, with the hazard ratios and corresponding 95% confidence intervals (CIs) being calculated. Statistical significance was set at *P* < 0.05. The boxplot and Kaplan–Meier survival curve of the filter lifespan were created using R-studio, version 4.2.1 (Integrated Development for R. RStudio, Inc., Boston, Massachusetts, USA).

### Ethics approval and consent to participate

This study was conducted with approval from the Ethics Committee of Hunan Children’s Hospital, approval number HCHLL-2023-192. The requirement for informed consent was waived.

## Results

### General status of the participants

**Figure 1:**
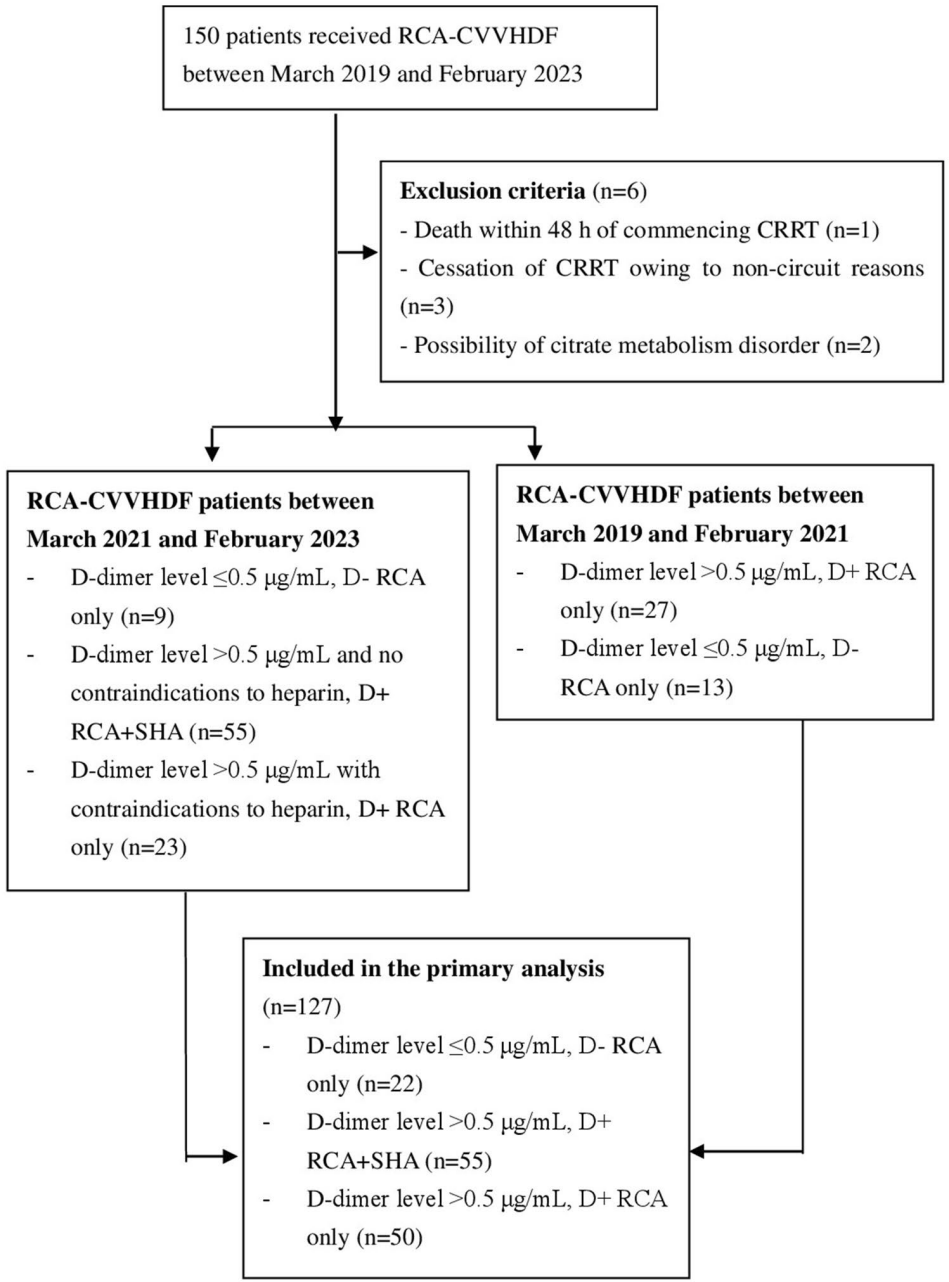
Flowchart of the patient selection process.

A total of 127 pediatric patients were included in this study. Figure 1 shows the flowchart of the study population selection process. Among the patients, 22, 50, and 55 patients belonged to the D− RCA only group, D+ RCA only group, and D+ RCA+ SHA group, respectively. In total, 52% of the patients received mechanical ventilation before CRRT, 33.9% required vasopressor support, and 82.7% had elevated D-dimer levels. The platelet count was lower in patients who received RCA only and was higher in those who underwent combination anticoagulation therapy. The most common indications for CRRT in descending order were sepsis, acute kidney injury, and cytokine storm. Table 1 presents the baseline characteristics of the patients.

**Table 1:** Baseline characteristics of participants before CRRT commencement.

Indicators	D− RCA only	D+ RCA only	D+ RCA+ SHA	*P* value
Number of patients	22	50	55	
Age in months [*M (P25, P75),* months]	35.5 (13.3, 53.8)	58 (12, 124.5)	33 (14, 103)	0.542
Sex (male/female, n)	16/6	30/20	36/19	0.573
Weight [*M (P25, P75),* kg]	12.5 (8.5, 18.1)	16.5 (11.0, 33.6)	13.0 (9.0, 24.5)	0.256
PCIS [*M (P25, P75),* points)]	73 (70, 76)	71 (66, 76)	72 (68, 76)	0.204
Mechanical ventilation [n (%)]	11 (50)	27 (54)	28 (50.9)	0.932
Vasopressor use [n (%)]	6 (27.3)	19 (38)	18 (32.3)	0.657
D-dimer level [*M (P25, P75)*, μg/mL]^a^	0.28 (0.18, 0.41)	2.15 (0.92, 7.40)	1.58 (1.08, 3.31)	< 0.001
PT [*M (P25, P75)*, s]	16.1 (14.7, 18.1)	15.7 (15.0, 17.9)	15.6 (13.6, 18.3)	0.841
aPTT [*M (P25, P75)*, s]	57.3 (51.8, 62.9)	55.0 (51.6, 60.0)	55.2 (50.3, 57.2)	0.184
Platelet count [*M (P25, P75)*, × 10^9^/L]^b^	57.5 (33, 228.5)	81.5 (44.3, 225.5)	162 (117, 287)	< 0.001
Hemoglobin level [*M (P25, P75)*, g/L]	88 (82.3, 112.3)	85 (77.8, 111.3)	92 (80, 118)	0.562
pH [*M (P25, P75)*]	7.28 (7.25, 7.29)	7.27 (7.24, 7.31)	7.27 (7.21, 7.29)	0.872
TBIL level [*M (P25, P75)*, mmol/L]	8.4 (5.3, 15.2)	8.1 (4.9, 16.1)	6.7 (4.3, 10.7)	0.255
HCO3- level (_x ± S, mmol/L)	22.1 ± 4.9	20.7 ± 4.2	20.5 ± 3.3	0.161
Creatinine level [*M (P25, P75)*, μmol/L]	26.1 (19.3, 96.3)	62.5 (33, 117.9)	66.6 (25.7, 108.6)	0.145
Serum calcium level [*M (P25, P75)*, mmol/L)]	2.35 (2.26, 2.42)	2.26 (2.12, 2.39)	2.33 (2.21, 2.45)	0.098
Systemic ionized calcium level [*M (P25, P75)*, mmol/L]	1.20 (1.11, 1.31)	1.22 (1.15, 1.27)	1.23 (1.13, 1.30)	0.937
Catheter location				0.565
Right internal jugular vein [n (%)]	9 (40.9)	27 (54.0)	26 (47.3)	
Femoral vein [n (%)]	13 (59.1)	23 (46.0)	29 (52.7)	
CRRT indications (n/%)				0.094
AKI	3 (13.6)	14 (28.0)	15 (27.3)	
Sepsis	6 (27.3)	16 (32.0)	14 (25.5)	
Acute poisoning	2 (9.1)	6 (12.0)	10 (18.2)	
Cytokine storm	6 (27.3)	12 (24.0)	5 (9.1)	
Fluid overload	5 (22.7)	2 (4.0)	11 (20.0)	

*PCIS* pediatric critical illness score, *PT* prothrombin time, *aPTT* activated partial thromboplastin time, *TBIL* total bilirubin, *CRRT* continuous renal replacement therapy, *AKI* acute kidney injury.

^a^D+ RCA only vs. D− RCA only, D+ RCA+ SHA vs. D− RCA only, both P < 0.05; D+ RCA only vs. D+ RCA+ SHA, *P* > 0.05.

^b^D− RCA only vs. D+ RCA+ SHA, D+ RCA only vs. D+ RCA+ SHA, both *P* < 0.05, D− RCA only vs. D+ RCA only, *P* > 0.05.

### Filter lifespan and bleeding episodes

**Figure 2:**
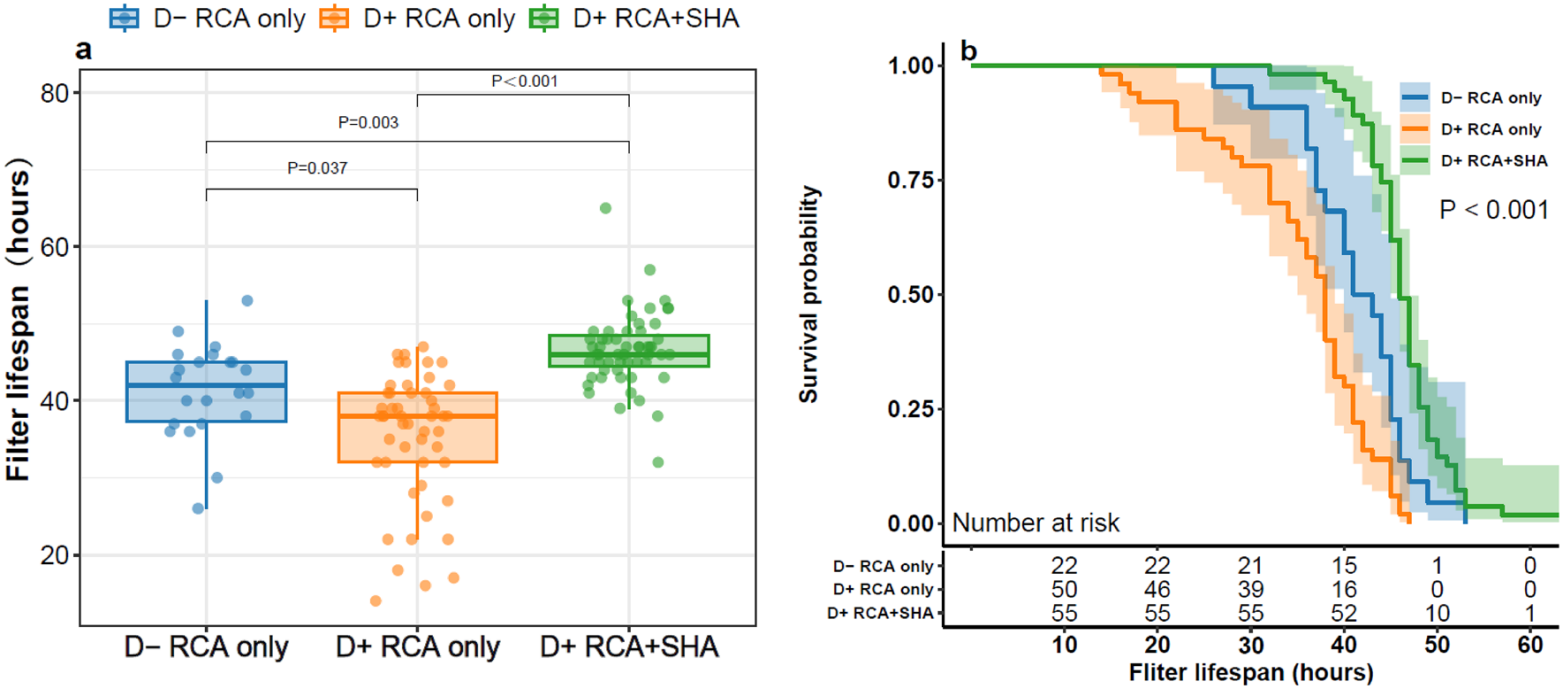
Filter lifespan. (**a**) Filter lifespan in the D− RCA only, D+ RCA only, and D+ RCA+ SHA groups shown as a boxplot with median and interquartile range. (**b**) Survival curves displaying filter lifespan among the D− RCA only, D+ RCA only, and D+ RCA+ SHA groups.

The filter lifespan was the longest in the D+ RCA+ SHA group and shortest in the D+ RCA only group (D- RCA only: 42.0 [37, 45.3] vs. D+ RCA only: 38.0 [32.0, 41.0] vs. D+ RCA+ SHA:46.0 [44.0, 49.0] h, *P* < 0.001), as shown in Fig. 2. In total, 43% (55/127) of the patients underwent SHA with RCA, and the incidence of bleeding episodes was not increased in these patients. There were no significant among-group differences in the incidence of bleeding episodes (D− RCA only: 18.2% vs. D+ RCA only: 18% vs. D+ RCA+ SHA: 14.5%, *P* = 0.869). Table 2 summarizes the filter characteristics and patient outcomes.

**Table 2:** CRRT-related indicators and outcomes.

Indicators	D− RCA only	D+ RCA only	D+ RCA+ SHA	*P* value
Number of patients	22	50	55	
Filtration fraction [*M (P25, P75),* %]*	19.2 (18.0, 19.6)	18.7 (17.6, 20.6)	19.5 (17.8, 21.7)	0.159
Blood flow rate (mL/min)*	3.1 (3.0, 3.5)	3.0 (2.9, 3.2)	3.0 (3.0, 3.3)	0.586
Citrate dose [*M (P25, P75),* mL/kg/h]*	4.7 (4.5, 5.4)	4.6 (4.4, 5.0)	4.7 (4.5, 5.3)	0.486
Effluent rate (mL/kg/h)*	44.8 (42.5, 51.0)	43.8 (41.8, 48.4)	45.4 (42.4, 50.4)	0.283
Mean post-filter ionized Ca level [*M (P25, P75)*, mmol/L]	0.33 (0.30, 0.34)	0.31 (0.30, 0.33)	0.32 (0.30, 0.33)	0.631
Post-filter ionized Ca level > 0.4 mmol/L [n (%)]	6 (27.3)	16 (32)	22 (40)	0.502
RBC transfusion during CRRT [n (%)]	10 (45.5)	16 (32.0)	16 (29.1)	0.378
Peak D-dimer level [*M (P25, P75)*, μg/mL]**	2.0 (1.2, 3.1)	6.9 (4.2, 13.9)	3.6 (2.5, 7.8)	< 0.001
Average D-dimer level [*M (P25, P75)*, μg/mL]**	1.2 (0.7, 1.9)	4.8 (2.8, 9.4)	2.2 (1.7, 3.2)	< 0.001
Peak hemoglobin level[*M (P25, P75)*, g/L]	89.0 (83.8, 112.3)	89.5 (81.8, 111.3)	92.0 (84.0, 118.0)	0.546
Average hemoglobin level [*M (P25, P75)*, g/L]	80.5 (78.6, 98.4)	83.2 (76.5, 102.3)	84.2 (76.8, 108.0)	0.580
Peak platelet [*M (P25, P75),* 100 × 10^9^/L]***	69 (53.5, 281.3)	99 (64.8, 240.5)	205 (161, 288)	< 0.001
Average platelet count [*M (P25, P75),* 100 × 10^9^/L]***	49.8 (34.4, 156.2)	78.6 (45.2, 182.1)	154.6 (127.0, 208.3)	< 0.001
RBC transfusion during CRRT [n (%)]	4 (18.2)	11 (22.0)	8 (14.5)	0.612
Filter lifespan [*M (P25, P75), h*]**	42.0 (37, 45.3)	38.0 (32.0, 41.0)	46.0 (44.0, 49.0)	< 0.001
Bleeding episodes [n (%)]	4 (18.2)	9 (18)	8 (14.5)	0.869
ICU stay [*M (P25, P75), d*]	12.5 (7, 20.8)	10 (6.8, 16.3)	11 (7, 22)	0.413
ICU mortality [n (%)]	2 (9.1)	7 (14)	6 (10.9)	0.806

*RBC* red blood cell, *CRRT* continuous renal replacement therapy.

*Time-weighted average.

**Pairwise comparisons among D− RCA only, D+ RCA only, and D+ RCA+ SHA, all P < 0.05.

***D− RCA only vs. D+ RCA only, *P* > 0.05; D− RCA only vs. D+ RCA+ SHA, D+ RCA only vs. D+ RCA+ SHA, both *P* < 0.05.

### Potential factors related to the risk of filter clotting

Results of univariate and multivariate Cox regression analysis revealed that concurrent heparin anticoagulation was an independent protective factor. Moreover, post-filter ionized calcium level > 0.4 mmol as well as elevated pre-CRRT hemoglobin and D-dimer levels were independent risk factors for filter clotting. Specifically, concurrent heparin anticoagulation was associated with a decreased risk of filter clotting (hazards ratio [HR] 0.116, 95% CI 0.074–0.183, P < 0.001); contrastingly, elevated pre-CRRT hemoglobin levels (HR 1.028, 95% CI 1.016–1.040, P < 0.001), elevated pre-CRRT D-dimer levels (HR 1.142, 95% CI 1.102–1.184, P < 0.001), and post-filter ionized calcium levels > 0.4 mmol/L (HR 2.046, 95% CI 1.278–3.275, P = 0.003) were associated with an increased risk of filter clotting (Table 3).

**Table 3:** Cox regression analysis of factors affecting filter lifespan.

Variable	Univariate analysis		Multivariate analysis	
	HR (95% CI)	P-value	HR (95% CI)	P-value
Concurrent heparin use	0.297 (0.203–0.434)	< 0.001	0.116 (0.074–0.183)	< 0.001
Post-filter ionized calcium level (> 0.4 mmol/L vs. ≤ 0.4 mmol/L)	2.006 (1.363–2.951)	< 0.001	2.046 (1.278–3.275)	0.003
Pre-CRRT hemoglobin level	1.023 (1.014, 1.033)	< 0.001	1.028 (1.016–1.040)	< 0.001
RBC transfusion during CRRT	1.367 (0.867–2.158)	0.179		
Pre-CRRT D-dimer level	1.121 (1.091–1.152)	< 0.001	1.142 (1.102–1.184)	< 0.001
Pre-CRRT platelet count	1.002 (1.001–1.003)	0.043	1.000 (0.998–1.001)	0.765

*CRRT* continuous renal replacement therapy, *RBC* red blood cell.

## Discussion

In this retrospective single-center cohort study, we compared the risk of filter clotting and incidence of bleeding episodes among the patients who underwent different anticoagulation therapies during CRRT. Our main finding was that the adoption of the combination of RCA and low-dose heparin anticoagulation strategy during CRRT may prolong the filter lifespan without increasing the incidence of bleeding episodes in patients with elevated D-dimer levels. Therefore, combination anticoagulation therapy may be superior to RCA alone.

Anticoagulation therapy is of utmost importance for preventing clotting in the extracorporeal circuit during CRRT. Various anticoagulation strategies, including SHA, RCA, nafamostat mesylate, low-molecular-weight heparin, argatroban, prostaglandin I2, and regional unfractionated heparin, are available for CRRT and have been extensively studied^12–18^. A meta-analysis that included 37 randomized controlled studies and 2648 participants revealed that heparin and citrate are the most commonly used anticoagulants during CRRT. Compared with heparin, RCA can effectively prolong the CRRT filter lifespan and reduce the risk of bleeding^2^. Recent randomized controlled studies have recommended the preferential selection of RCA over SHA in patients undergoing CRRT since it allows for a relatively longer filter lifespan^5,19^. However, the pre-thrombotic state was not considered in these studies. Accordingly, suitable anticoagulation strategies for managing patients with potential hypercoagulability due to elevated D-dimer levels prior to CRRT remain unclear.

Since 2017, RCA has been preferentially selected at our unit for most patients requiring CRRT who lack contraindications to citrate. This has led to improvements in the CRRT anticoagulation effects and filter lifespan. However, in our clinical practice, we have also observed that the expected filter lifespan was not reached in certain pediatric patients with elevated D-dimer levels who underwent RCA-CRRT. D-dimer levels are associated with potential thrombosis and can effectively predict thrombotic complications^20,21^. Elevated D-dimer levels may be associated with an increased risk of thrombosis^22^, an increased tendency for CRRT filter clotting, and a shortened filter lifespan. These D-dimer levels have been identified as a risk factor for CRRT filter clotting^10^; however, such a condition may be improved with heparin administration^23^. Based on the principles of anticoagulation, the concurrent use of heparin and citrate anticoagulation to exert effects on different pathways of the coagulation cascade may serve as a good anticoagulation strategy during CRRT for managing patients with elevated D-dimer levels. Ionized calcium acts as Factor IV in the coagulation cascade, and citrate can inhibit the coagulation process through reversible chelation of ionized calcium in the blood. Heparin can bind to antithrombin III (AT III) by inducing conformational changes in Factor Xa, considerably enhancing AT III activity, which leads to the inhibition of Factor Xa and Factor IIa activation in the coagulation cascade and ultimately produces an anticoagulant effect^24^. Therefore, the synergistic effect of citrate and heparin may be utilized to prolong the CRRT filter lifespan. However, there have been limited studies on the adoption of a combination citrate and heparin anticoagulation. Currently available research has focused on patients with COVID-19 who experienced premature extracorporeal clotting during CRRT due to a hypercoagulable state and involved the addition of heparin to citrate for the prolongation of the filter lifespan^10,11,25,26^. Valle et al.^10^ reported that the CRRT filter lifespan in patients with COVID-19 was associated with D-dimer levels, with the risk of filter clotting increasing 0.94 times when D-dimer levels were ≥ 5990 ng/mL. The median filter lifespan was 25.6 h in patients with COVID-19 who underwent citrate anticoagulation only; however, it considerably increased to 81.9 h in patients with COVID-19 who underwent combination anticoagulation therapy with citrate and heparin. Additionally, the risk of filter clotting was decreased by 0.37 times with the addition of heparin. These previous findings are consistent with our findings. Specifically, the CRRT filter lifespan was 42.0 (37, 45.3) h, 38.0 (32.0, 41.0) h, and 46.0 (44.0, 49.0) h in the D− RCA only, D+ RCA only, and D+ RCA+ SHA groups, respectively. Therefore, systemic anticoagulation involving the addition of low-dose heparin to RCA may reduce the coagulation risk and prolong the CRRT filter lifespan in patients with elevated D-dimer levels.

Our findings indicated that the addition of low-dose heparin for systemic anticoagulation did not increase the incidence of bleeding episodes or demand for RBC transfusion in patients undergoing CRRT, which is consistent with the findings reported by Valle et al.^10^. This may be attributed to the relatively low heparin dose adopted in our study.

The CRRT filter lifespan is affected by multiple factors. For example, high ionized calcium levels are associated with a decreased filter lifespan^27^. In our study, the risk of filter clotting was increased when the post-filter ionized calcium level exceeded 0.4 mmol/L. Moreover, hemoglobin levels were negatively correlated with the filter lifespan, which is consistent with the findings in adult studies^10^. Approximately 33–65% of patients undergoing CRRT require blood transfusions^28–30^. Our results indicated that the CRRT filter lifespan was not associated with increased RBC transfusion, which is consistent with the findings reported by a case–control study^28^. A prospective study including pediatric patients undergoing RCA-CRRT demonstrated that both direct blood transfusion during CRRT and partial replacement of citrate transfusion (i.e., adoption of a reduced citrate infusion dose during blood transfusion in RCA-CRRT) were not associated with filter clotting during RCA-CRRT^31^. This could be attributed to the fact that the citrate content in blood products increases the total citrate dose for CRRT anticoagulation. This further reduces ionized calcium levels in the extracorporeal circuit, and thereby maintains effective anticoagulation without increasing the risk of filter clotting. Multivariate Cox regression analysis further revealed that the platelet count did not affect the CRRT filter lifespan. There remains no consensus on the effect of the platelet count on the filter lifespan. Certain studies have reported a negative correlation of the CRRT filter lifespan with a lower platelet count^32^, with the risk of filter clotting increasing when the platelet count exceeds 45 × 10^9^/L^33^. However, another study has suggested a lack of association between platelet count and filter lifespan^34^.

To date, this is the largest study comparing between the anticoagulation strategies of RCA alone and RCA combined with SHA in patients other than those with COVID-19. Additionally, it is the first study of its kind in a pediatric population.

Our study has limitations. First, this was a single-center retrospective observational study with a limited sample size. CRRT initiation was determined solely at the discretion of attending physicians in the PICU, which may limit the universal applicability of our results. Second, D-dimer levels were used to assess the presence or absence of a hypercoagulable state. Furthermore, D-dimer levels are influenced by various factors and may not comprehensively and accurately reflect the pre-thrombotic state. Therefore, caution should be exercised when interpreting the study results. Finally, patients who underwent combination anticoagulation therapy in this study were not stratified based on D-dimer levels. Therefore, the optimal D-dimer level for the adoption of RCA combined with SHA remains unclear, and our results may not accurately reflect the relationship between D-dimer levels and the CRRT filter lifespan.

## Conclusions

RCA combined with low-dose heparin anticoagulation during CRRT could reduce the risk of filter clotting and prolong the filter lifespan without increasing the risk of bleeding in patients at a high risk of hypercoagulability due to elevated D-dimer levels.

## Data Availability

All data generated or analyzed during this study are included in this published article. Further inquiries can be directed to the corresponding author.
